# Implementing Systematic Patient-Reported Measures for Chronic Conditions Through the Naveta Value-Based Telemedicine Initiative: Observational Retrospective Multicenter Study

**DOI:** 10.2196/56196

**Published:** 2024-06-28

**Authors:** Gabriel Mercadal-Orfila, Salvador Herrera-Pérez, Núria Piqué, Francesc Mateu-Amengual, Pedro Ventayol-Bosch, María Antonia Maestre-Fullana, Joaquín Ignacio Serrano-López de las Hazas, Francisco Fernández-Cortés, Francesc Barceló-Sansó, Santiago Rios

**Affiliations:** 1 Pharmacy Department Hospital Mateu Orfila Mahó Spain; 2 Department of Biochemistry and Molecular Biology Universitat de les Illes Balears Mallorca Spain; 3 Facultad de Ciencias de la Salud Universidad Internacional de Valencia Valencia Spain; 4 CIBER Epidemiología y Salud Pública (CIBERESP) Barcelona Spain; 5 Microbiology Section, Department of Biology, Healthcare and Environment Faculty of Pharmacy and Food Sciences Universitat de Barcelona Barcelona Spain; 6 Research Institute of Nutrition and Food Safety (INSA-UB) Universitat de Barcelona Barcelona Spain; 7 Healthcare Industry Solutions at MongoDB - Digital Health & Innovation Barcelona Spain; 8 Pharmacy Department Hospital Universitari Son Espases Palma de Mallorca Spain; 9 Pharmacy Department Hospital de Manacor Manacor Spain; 10 Pharmacy Department Hospital Universitari Son Llàtzer Palma de Mallorca Spain; 11 Pharmacy Department Hospital Comarcal d’Inca Inca Spain; 12 Pharmacy Department Hospital Can Misses Eivissa Spain; 13 Departament de Genètica, Microbiologia i Estadística Facultat de Biologia Universitat de Barcelona Barcelona Spain

**Keywords:** chronic conditions, eHealth, value-based care, patient-reported outcome measures, patient-reported experience measures, questionnaires, response rate, telemedicine platform

## Abstract

**Background:**

Patient-reported outcome and experience measures can play a critical role in providing patient-centered and value-based health care to a growing population of patients who are chronically ill. Value-based telemedicine platforms such as the Naveta initiative may facilitate the effective integration of these tools into health care systems.

**Objective:**

This study aims to evaluate the response rate to electronic patient-reported outcome measures (ePROMs) and electronic patient-reported experience measures (ePREMs) among patients participating in the Naveta telemedicine initiative and its correlations with sociodemographic and clinical characteristics, as well as the evolution of the response rates over time.

**Methods:**

Between January 1, 2021, and June 30, 2023, a total of 53,364 ePREMs and ePROMs for 20 chronic conditions were administered through the Naveta-Phemium platform. Descriptive statistics were used to summarize continuous and categorical variables. Differences in response rates within each sociodemographic variable were analyzed using logistic regression models, with significance assessed via chi-square and post hoc Tukey tests. Two-way ANOVA was used to examine the interaction between time interval and disease type on response rate evolution.

**Results:**

A total of 3372 patients with severe chronic diseases from 64 public hospitals in Spain participated in the Naveta health questionnaire project. The overall response rate to ePROMs and ePREMs during the first 2.5 years of the Naveta initiative was 46.12% (24,704/53,364), with a baseline rate of 53.33% (7198/13,496). Several sociodemographic factors correlated with lower response rates, including male gender, older age, lower education level, frequent alcohol use, being a student, and not being physically active. There were also significant variations in response rates among different types of chronic conditions (*P*<.001), with the highest rates being for respiratory (433/606, 71.5%), oncologic (200/319, 62.7%), digestive (2247/3601, 62.4%), and rheumatic diseases (7506/12,982, 57.82%) and the lowest being for HIV infection (7473/22,695, 32.93%). During the first 6 months of follow-up, the response rates decreased in all disease types, except in the case of the group of patients with oncologic disease, among whom the response rate increased up to 100% (6/6). Subsequently, the overall response rate approached baseline levels.

**Conclusions:**

Recognizing the influence of sociodemographic factors on response rates is critical to identifying barriers to participation in telemonitoring programs and ensuring inclusiveness in patient-centered health care practices. The observed decline in response rates at follow-up may be due to survey fatigue, highlighting the need for strategies to mitigate this effect. In addition, the variation in response rates across chronic conditions emphasizes the importance of tailoring telemonitoring approaches to specific patient populations.

## Introduction

### Background

Health care systems and institutions worldwide are undergoing transformation to meet the demands of an aging population, implement new health care standards, and integrate advancements in digital technology [1-4]. Increased life expectancy and improvements in medicine have led to a growing number of people living with chronic conditions [5-7]. This growth represents a major challenge for health care systems, which must provide efficient care for patients with chronic conditions while wisely allocating limited resources [3,8]. To address these complex challenges, governments, health organizations, and health care providers are increasingly recognizing the need to transition toward more personalized and patient-centered care models, aligned with the concept of value-based health care (VBHC) [9-12]. In this new paradigm, value is defined as health outcomes that matter to patients in comparison to the cost of achieving these outcomes [9].

To carry out these changes, health care services and systems need tools to guide the transition [11,13]. Measuring, reporting, and comparing outcomes is essential to assess the value of care provided to patients [9,14,15]. Patient-reported measures are the fundamental tools used to guide health systems and providers in implementing person-centered health care and achieving outcomes that matter to patients [16,17]. Patient-reported measures are administered in the form of questionnaires and include patient-reported outcome measures (PROMs) and patient-reported experience measures (PREMs). PROMs can assess a variety of outcomes, such as physical performance, social functioning, psychological well-being, symptom severity, disability, and impairment, from the perspective of the patient, whereas PREMs focus on the patient’s experience of care [18]. These measures can be used to support diagnosis, monitor treatment and patient progress, improve communication between patient and health care professionals, and facilitate shared decision-making [17,19]. Despite their potential value and some examples of successful health system–level PREM and PROM programs, such as the National Health Service PROMs program in the United Kingdom and the Danish patient-reported outcome (PRO) system, their routine use in health systems is not widespread in other countries [11,13,16,18,20-22].

### The Naveta Value-Based Telemedicine Initiative

Digital transformation and the integration of eHealth tools and value-based IT platforms in health care settings are critical for achieving the systematic delivery of standardized PREMs and PROMs in clinical practice [11,23,24]. The COVID-19 pandemic has sparked a revolution in digital health technologies that can pave the way for this transformation [4,25]. In this context, the Naveta initiative [26] has emerged as a scientific community that aims to promote VBHC for patients with serious chronic conditions by implementing processes and strategies related to the electronic delivery of PROMs and PREMs and the use of telemedicine tools.

Naveta was created by the Association of Pharmacists of Outpatient Departments of the Balearic Islands (FARUPEIB) with the participation of BiblioPRO, a web-based library of PRO questionnaires in Spanish [27,28]. The technological infrastructure that supports Naveta activities is provided by Phemium [29], a software platform specialized in telemedicine projects. The scientific committee of Naveta is currently composed of physicians, hospital pharmacists, nurses, psychologists, members of BiblioPRO, and experts in eHealth and innovation. The committee’s responsibilities include selecting PROM standard sets, determining the frequency and timing of questionnaire administration, evaluating and improving the efficiency of the platform and standard sets, promoting VBHC, mentoring and advising health care professionals and researchers, and fostering new technological developments within the Naveta ecosystem. To date, specific and generic PROM standard sets have been selected for 20 chronic diseases. These measures can assess, among other outcomes, health status, disease activity, impact on quality of life, treatment satisfaction, and productivity from the patient’s perspective.

**Figure 1:**
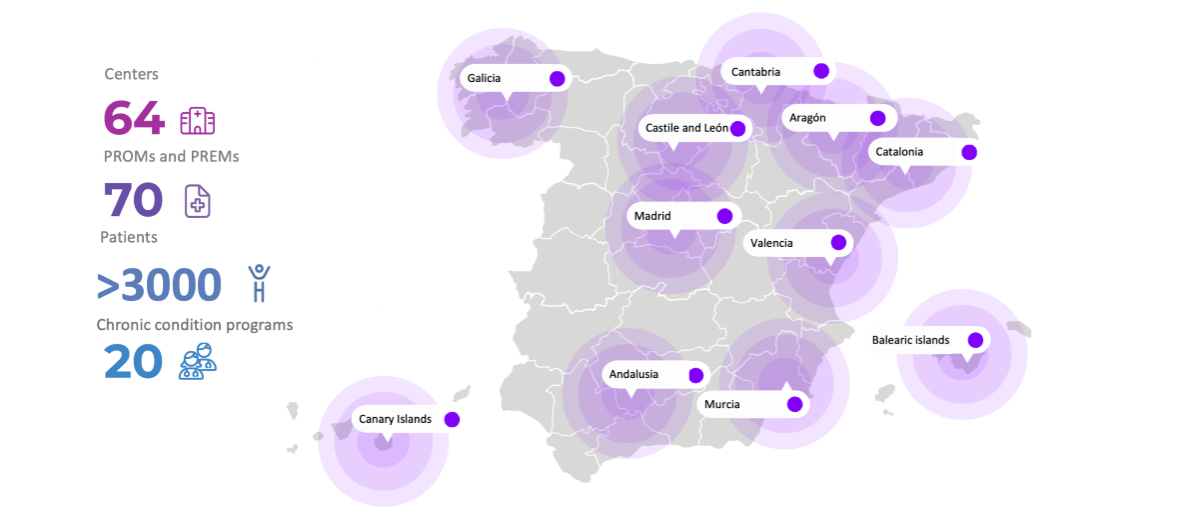
Distribution of Spanish hospitals participating in the Naveta initiative. PREM: patient-reported experience measure; PROM: patient-reported outcome measure.

One of the goals of the Naveta initiative is to provide state-of-the-art PROM and PREM tools that enable health care managers and professionals to obtain accurate and valuable information directly from patients to be incorporated into the decision-making process. Another goal of the initiative is to build a body of knowledge on the appropriate use of PROMs, considering both disease type and sociodemographic data, in line with initiatives such as Patient-Reported Outcomes Tools: Engaging Users and Stakeholders (PROTEUS) and the International Consortium for Health Outcomes Measurement (ICHOM) [30,31]. Recognized recently by different Spanish associations as an outstanding innovation project, Naveta is one of the most widespread value-based telemedicine initiatives in Spain [32] and has been widely implemented at hospitals and institutions in different Spanish regions (Figure 1). Furthermore, several observational studies on patients with various chronic diseases are currently being conducted using the Naveta VBHC approach.

### Goal of the Study

Here, we present our experience in implementing the Naveta value-based telemedicine initiative at different hospitals in Spain, which involved the administration of 53,634 electronic PROMs (ePROMs) and electronic PREMs (ePREMs) to 3372 patients with severe chronic conditions over the last 2.5 years.

The primary objective of this study was to assess the response rate to electronic questionnaires among patients who were chronically ill registered in Naveta and to examine its correlation with various sociodemographic characteristics. In addition, this study aimed to track the evolution of response rates over time, specifically among patients with different types of chronic diseases. The purpose of this analysis was to identify potential biases in the collection of information that might have prevented the comprehensive monitoring of the maximum number of patients.

## Methods

### Study Design and Sample Selection

This study was carried out in the context of routine clinical practice in which patients were receiving dual care (on-site plus telematic care with the Naveta platform). We conducted an observational retrospective multicenter study involving patients with severe chronic diseases. These patients were regularly receiving hospital-only medicines at pharmacy outpatient clinics of 64 public hospitals in different regions of Spain (Figure 1). Patients were enrolled at the time of starting or changing their prescribed outpatient medication and registered for ≥1 chronic condition programs based on their diagnosis. The study covered the period from January 1, 2021, to June 30, 2023.

All patients met the following criteria: aged ≥18 years, diagnosed with ≥1 chronic diseases (psoriasis, psoriatic arthritis, rheumatoid arthritis, asthma, cancer, ulcerative colitis, atopic dermatitis, Crohn disease, multiple sclerosis, ankylosing spondylitis, hidradenitis suppurativa, urticaria, and HIV infection, among others), and agreed to participate in a telemedicine project linked to health questionnaires through the Naveta-Phemium platform.

### Selection of Patient-Reported Measures

**Figure 2:**
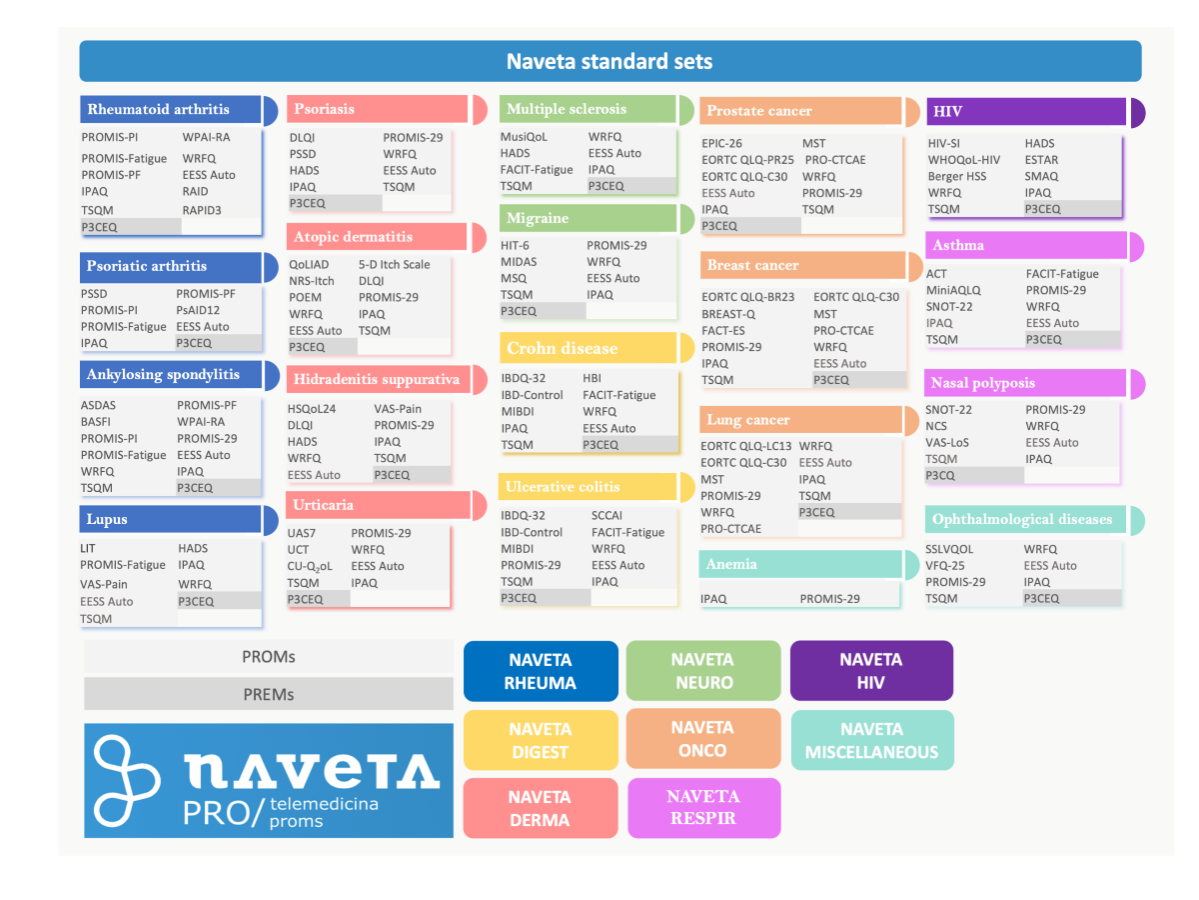
Naveta’s standard sets of patient-reported measures for 20 chronic conditions. ACT: Asthma Control Test; Ankyl: ankylosing; ARDs: Autoimmune Rheumatoid Diseases; ASDAS: Ankylosing Spondylitis Disease Activity Score; BASFI: Bath Ankylosing Spondylitis Functional Index; Berger HSS: Berger HIV Stigma Scale; CU-Q2oL: Chronic Urticaria Quality of Life Questionnaire; DLQI: Dermatology Life Quality Index; EESS auto: self-reported side effects; EORTC: European Organization for Research and Treatment of Cancer; EORTC QLQ-BR23: EORTC Quality of Life Questionnaire for Breast Cancer; EORTC QLQ-C30: EORTC Core Quality of Life questionnaire; EORTC QLQ-LC13: EORTC Quality of Life Questionnaire module for Lung Cancer patients; EORTC QLQ-PR25: EORTC Quality Of Life Questionnaire Module For Prostate Cancer 25; EPIC-26: Expanded Prostate Cancer Index Composite-26; FACIT-Fatigue: Functional Assessment of Chronic Illness Therapy – Fatigue Scale; FACT-ES: Functional Assessment of Cancer Therapy for patients with Endocrine Symptoms; HADS: Hospital Anxiety and Depression Scale; HBI: Harvey-Bradshaw Index; Hidra.: Hidradenitis; HIT-6: Headache Impact Test -6; HSQoL24: Hidradenitis Suppurativa Quality of Life 24; HIV SI: HIV Symptom Index; IBD Control: Inflammatory Bowel Disease Control Questionnaire; IBDQ32: Inflammatory Bowel Disease Questionnaire; IPAQ: International Physical Activity Questionnaire; LIT: Lupus Impact Tracker; MIBDI: Manitoba Inflammatory Bowel Disease Index; MIDAS: Migraine Disability Assessment; mini AQLQ: Mini Asthma Quality of Life Questionnaire; MSQ: Migraine-Specific Quality-of-Life Questionnaire; MST: Malnutrition screening tool; MusiQoL: multiple sclerosis international quality of life; NCI’s PRO-CTCAE: National Cancer Institute’s Patient-Reported Outcomes version of the Common Terminology Criteria for Adverse Events; NCS: Nasal Congestion Score; NRS: Numeric Rating Scale; Ophtalmol: Ophthalmological; PROMIS: Patient Reported Outcome Information System; PROMIS-PF: PROMIS Physical Function; PROMIS-PI: PROMIS Pain Intensity; PsAID12: 12-item Psoriatic Arthritis Impact of Disease; PSSD: Psoriasis Symptoms and Signs Diary; P3CEQ; Person-Centred Coordinated Care Experience Questionnaire; QoLIAD: Quality of Life Index for Atopic Dermatitis; RAID: Rheumatoid Arthritis Impact of Disease; RAPID3: Routine assessment of patient index data 3 ; SCCAI: Simple Clinical Colitis Activity Index; SLVQOL: Spanish low vision quality of life questionnaire; SMAQ: Simplified Medication Adherence Questionnaire; SNOT-22: Sino-nasal outcome test 22; TSQM: Treatment Satisfaction Questionnaire for Medication; UAS7: Urticaria activity Score 7; UCT: Urticaria Control Test; VAS: Visual Analogue Scale; VAS-LoS: VAS for loss of smell; VFG-25: Visual Functioning Questionnaire 25; WHOQoL–HIV: World Health Organization’s Quality of Life HIV instrument; WPAI-RA: Work Productivity in Rheumatoid Arthritis; WRFQ: Work Role Functioning Questionnaire.

The questionnaires included in each disease standard set, selected by Naveta’s scientific committee, are specified in Figure 2. The color code in the figure indicates the type of medical condition: rheumatic diseases, gastrointestinal diseases, neurological diseases, skin diseases, oncologic diseases, respiratory diseases, HIV infection, and other diseases.

The scientific committee that selects Naveta’s standard sets of PROMs for each pathology is composed of physicians, hospital pharmacists, psychologists, members of BiblioPRO, and eHealth experts.

Their decisions are informed by the study of literature reviews and recommendations from authoritative sources such as the Patient-Reported Outcomes Measurement Information System (PROMIS) [33], the ICHOM initiative [34], and the Patient-Reported Indicator Surveys initiative of the Organisation for Economic Co-operation and Development [35]. The questionnaires used are the validated Spanish versions and meet licensing requirements. The platform mainly included PROMS for a variety of chronic conditions (67), and only a few PREMs were administered, including the Person-Centered Coordinated Care Experience Questionnaire (P3CEQ), which is designed to assess person-centered coordinated care from the perspective of people with long-term conditions [36,37]. Other PREMs included the Self-Injection Assessment Questionnaire (SIAQ) [38,39] and a Likert scale survey to assess satisfaction with the dual follow-up (on-site and telematic care; administered annually).

### Data Collection Procedures

Patients who provided informed consent (refer to the Ethical Considerations subsection for details) received a link to access the electronic questionnaires, which they could complete on their own device (PC, tablet, or mobile phone). Each patient was provided with a standard set of PROMs tailored to their specific chronic disease. Participants were also asked to complete a sociodemographic questionnaire that included questions about their level of education, employment status, smoking habit and alcohol consumption, BMI, and physical activity. The BMI parameter was categorized according to the World Health Organization classification: underweight (<18.5 kg/m^2^), healthy weight (18.5-24.9 kg/m^2^), overweight (25.0-29.9 kg/m^2^), and obese (≥30 kg/m^2^) [40]. After 1 year of follow-up with the Naveta telemedicine program, patients were administered a 10-point Likert scale questionnaire to assess their satisfaction, and this survey was repeated annually.

**Figure 3:**
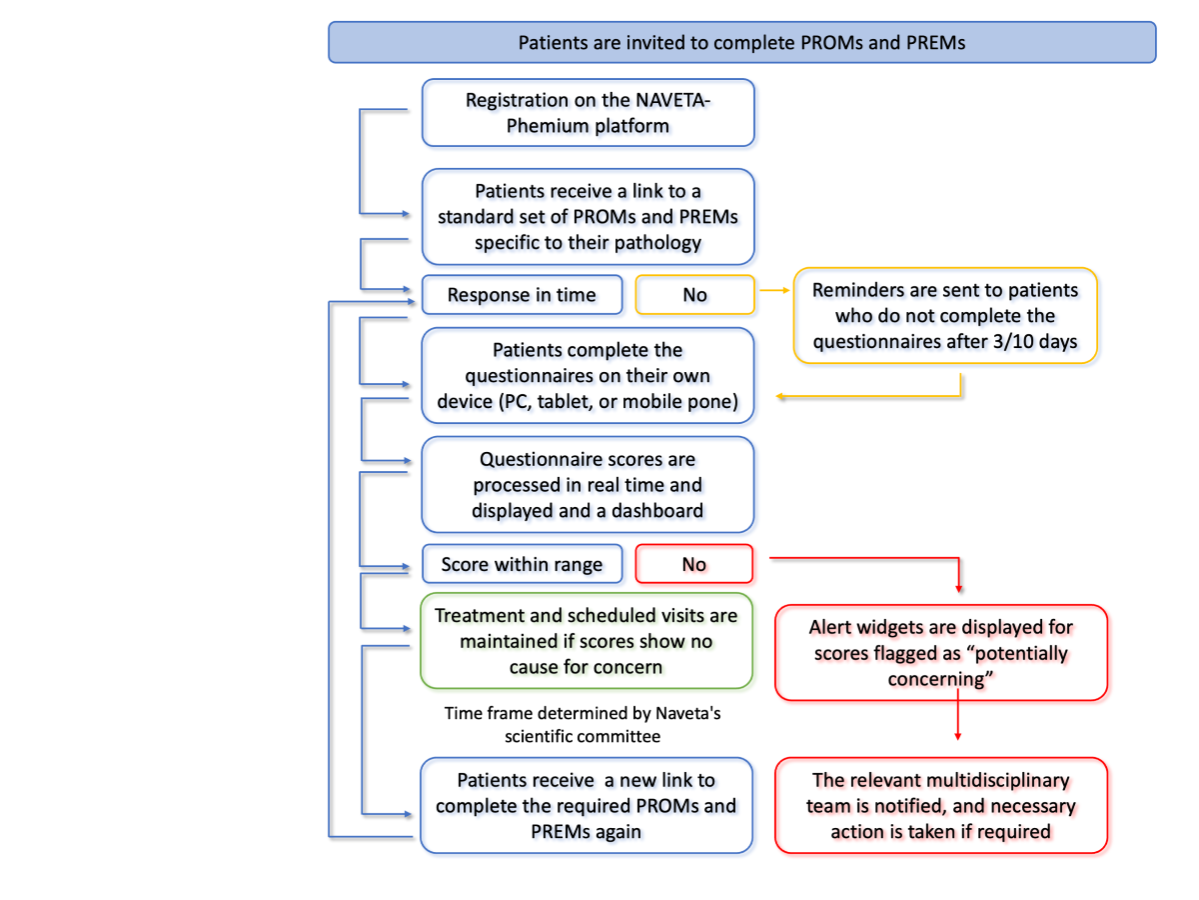
Process of delivering and collecting patient-reported measures through the Naveta-Phemium platform. PREM: patient-reported experience measure; PROM: patient-reported outcome measure.

The process of delivering and collecting PROMs and PREMs through the Naveta-Phemium platform is illustrated in Figure 3. The system automatically generates and sends reminders (via SMS text messaging and email) to patients who do not complete the questionnaires 7 and 15 days after receiving the link. It also generates alert widgets for potentially concerning scores and enables users to specify the score categories that should be flagged for each PROM (eg, scores indicating moderate to severe impairment). These alerts, along with other data from the patient’s medical record, are used to make clinical decisions about medication changes, specialist referrals, lifestyle recommendations, and more. The patients were requested to complete the relevant standard set of questionnaires at the start of each pharmacological treatment or at each medication change (baseline collection). The periodicity of the follow-up questionnaires was determined by the Naveta scientific committee according to the specific chronic conditions and the outcome measured.

The team at each center was responsible for seeking permission to use the questionnaires through the Naveta initiative.

### Technology Infrastructure

The Naveta initiative is supported by Phemium [29], a technological multiplatform specialized in the design and implementation of telemedicine projects. This platform has >2000 active professional users in different countries (Spain, Poland, the Czech Republic, Germany, Chile, Peru, Mexico, and Nigeria), with >2 million health care interactions per year globally. It complies with the requirements of a high standard of data protection, level 3, according to the European General Data Protection Regulation and Spanish Organic Law 3/December 5, 2018, regarding the protection of personal data and the guarantee of digital rights.

**Figure 4:**
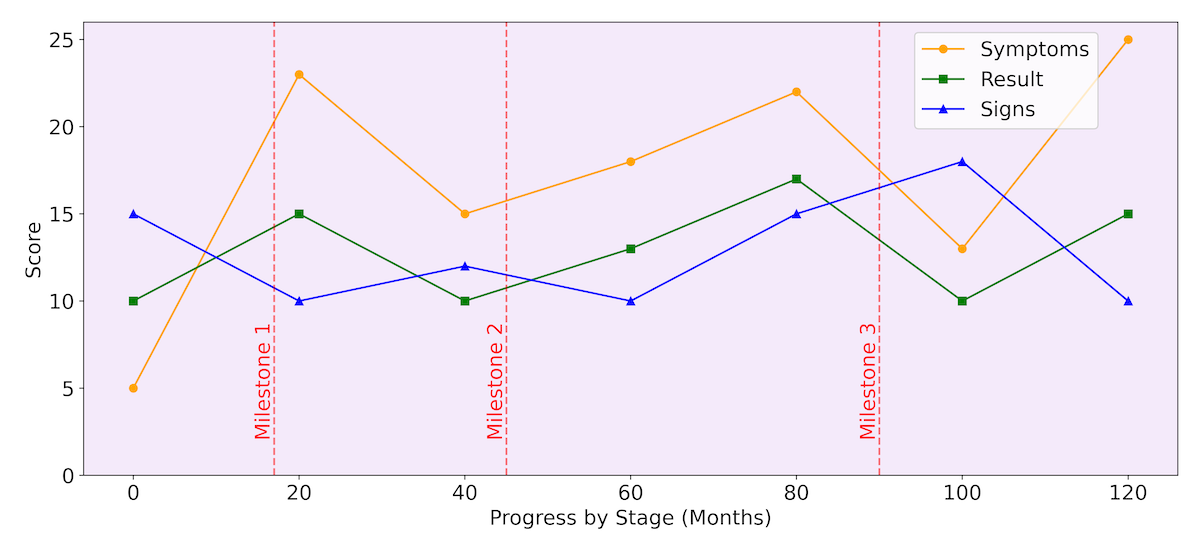
Psoriasis Symptoms and Signs Diary: an example of a patient-reported outcome measure dashboard. This dashboard monitors 3 distinct metrics—symptoms, results, and signs—over a 120-month time frame. Notably, it features milestone markers at roughly 17, 45, and 90 months, with dashed vertical lines denoting significant events in the management of the condition, such as weight loss, changes in alcohol consumption, or alterations in treatment, among potential examples.

The platform facilitates effective communication between health care professionals and patients. To systematize the delivery and interpretation of PROMs and PREMs in health care practice, the system performs the parameterization and customization of protocols. Each PROM is labeled for ≥1 specific conditions or as a generic questionnaire. The system interprets patient responses according to the score intervals of each questionnaire. Responses that do not meet the conditions required for a particular questionnaire are automatically discarded. The results of the questionnaires are processed in real time and presented graphically on a dashboard (Figure 4). This allows health care professionals (nurses, hospital pharmacists, and physicians) to immediately monitor patient outcomes and compare their progress with that of other patients and to monitor the evolution of an individual patient in a clinical context. The system also allows global analysis within groups for each pathology and compares individual and global results. In addition, patients can access their results on their dashboard.

### Statistical Analysis

Descriptive statistics were used to summarize continuous variables as means and SDs and categorical variables as frequencies and percentages. For results analysis, chronic conditions were categorized based on their respective disease type (Figure 2). To assess the response rates over time, the number of completed questionnaires at distinct intervals was calculated. Differences in response rates among categories within each sociodemographic variable were analyzed using logistic regression models. Their significance was evaluated with chi-square and post hoc Tukey tests (using the *glht* function from the *multcomp* R package). A 2-way ANOVA was used to study the interaction effect between time interval and disease type on response rate evolution. All statistical procedures were performed using R software (version 4.2.2; R Foundation for Statistical Computing) [40], and a significance level of *P*≤.05 was set for all tests.

### Ethical Considerations

Ethics approval was obtained from the research ethics committee of the Balearic Islands (Comitè d’ètica de la investigació de les Illes Balears; IB 5117/23 EOm). This study was conducted in accordance with the principles of the Declaration of Helsinki and its subsequent revisions. The informed consent form and other patient information were reviewed and approved by the clinical research ethics committee and the independent ethics committee of Balearic Islands (Palma de Mallorca, Spain).

Before enrollment in the Naveta project, all patients received detailed information and provided written informed consent.

## Results

### Descriptive Analysis of the Sample

**Figure 5:**
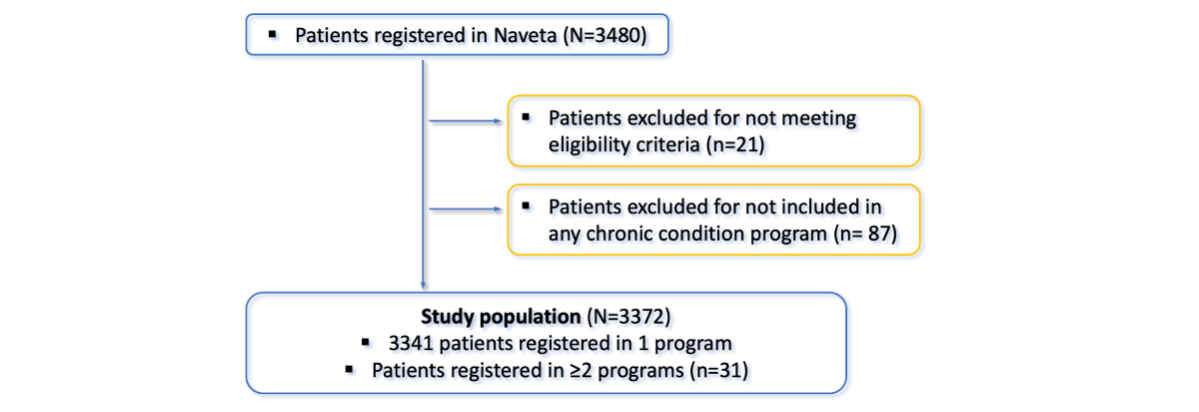
Flowchart for participant recruitment.

In total, 3372 patients diagnosed with at least 1 chronic condition were registered on the Naveta-Phemium platform: 2128 (63.11%) men, 1234 (36.6%) women, and 10 (0.3%) people who self-identified as *other gender*. Of the 3372 patients, 3341 (99.08%) were registered in only 1 chronic condition program, while 31 (0.92%) had multiple diagnoses and were registered in ≥2 chronic condition programs (n=28, 90% were registered in 2 programs; n=2, 6% in 3 programs; and n=1, 3% in 4 programs; Figure 5). The mean age of the whole cohort was 50.36 (SD 12.91) years.

**Figure 6:**
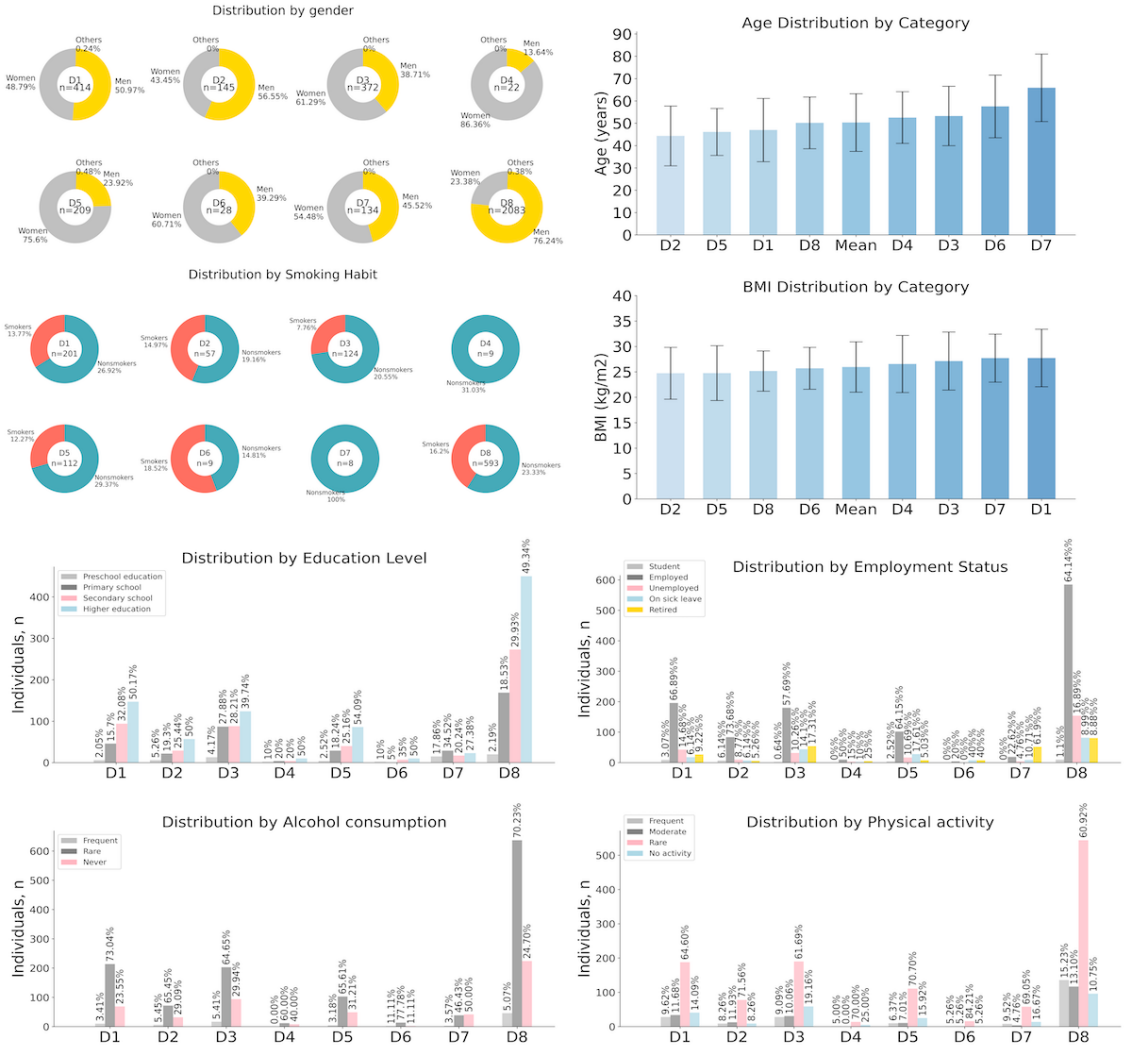
Sociodemographic characteristics of study participants by disease type (a comprehensive analysis of demographic distribution and health habits across different disease categories). The charts illustrate the distribution—among individuals with dermatological, digestive, respiratory, neurological, oncologic, HIV-related, and miscellaneous conditions—by (A-D) gender, age, BMI, and smoking habits; and (E-H) education level, employment status, alcohol consumption, and physical activity. D1: dermatological diseases; D2: digestive diseases; D3: rheumatological diseases; D4: respiratory diseases; D5: neurological diseases; D6: oncological diseases; D7: miscellaneous diseases; D8; HIV.

The HIV disease program had the highest number of registered patients (2083/3372, 61.77%), whereas the breast cancer (17/3372, 0.5%), prostate cancer (9/3372, 0.27%) and lung cancer (2/3372, 0.06%) programs had the lowest number of registered patients (Table S1 in Multimedia Appendix 1). Within the disease type categories, the largest group was Naveta HIV (2083/3372, 61.77%), followed by Naveta Derma (414/3372, 12.28%), Naveta Rheuma (372/3372, 11.03%), Naveta Miscellaneous (134/3372, 3.97%), Naveta Neuro (209/3372, 6.2%), Naveta Digest (145/3372, 4.3%), Naveta Onco (28/3372, 0.83%), and finally Naveta Respir (22/3372, 0.65%). Figure 6 and Table S2 in Multimedia Appendix 1 present the main sociodemographic characteristics of the study participants by type of disease.

### Descriptive Analysis of the Questionnaires

**Figure 7:**
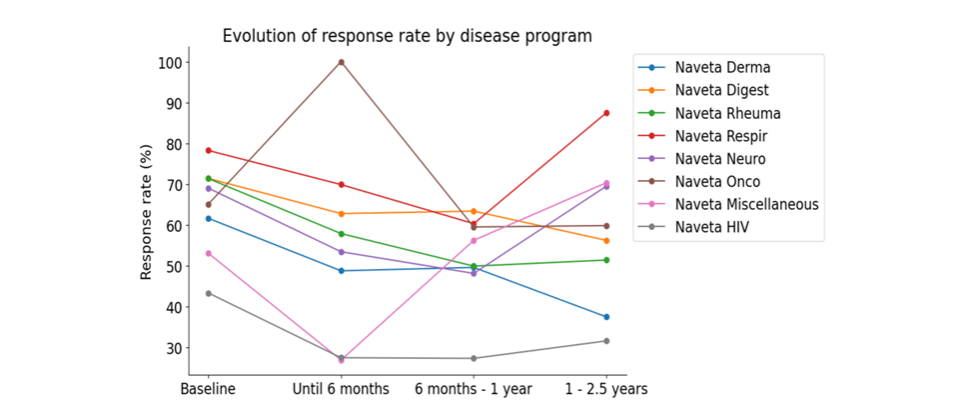
Evolution of response rate by disease program.

**Figure 8:**
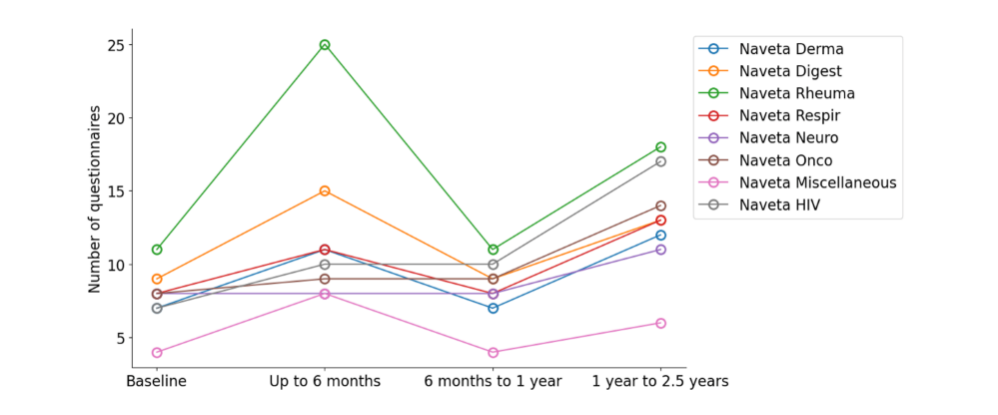
Number of questionnaires sent out by disease program.

A total of 53,364 questionnaires were sent out for all disease categories, and 24,704 (46.12%) were completed. The response rates (percentage of completed questionnaires out of the total number of questionnaires sent) at specific time intervals were as follows: 53.33% (7198/13,496) at baseline, 41.5% (9131/22,000) from baseline to 6 months, 43.7% (4474/10,239) from 6 months to 1 year, and 51.13% (3901/7629) from 1 year to 2.5 years (Figure 7; Table S3 in Multimedia Appendix 1). The analysis revealed a significant interaction effect between time interval and disease type on response rate evolution over time (*P*<.001). In all disease categories, the response rate decreased after 6 months, except in the category of oncologic diseases, where the response rate increased up to 100% (6/6). In the following intervals, the evolution of the response rate did not show a homogeneous trend in the different disease categories. It should be noted that the number of questionnaires sent to patients for completion differed based on the type of disease (Figure 8).

**Figure 9:**
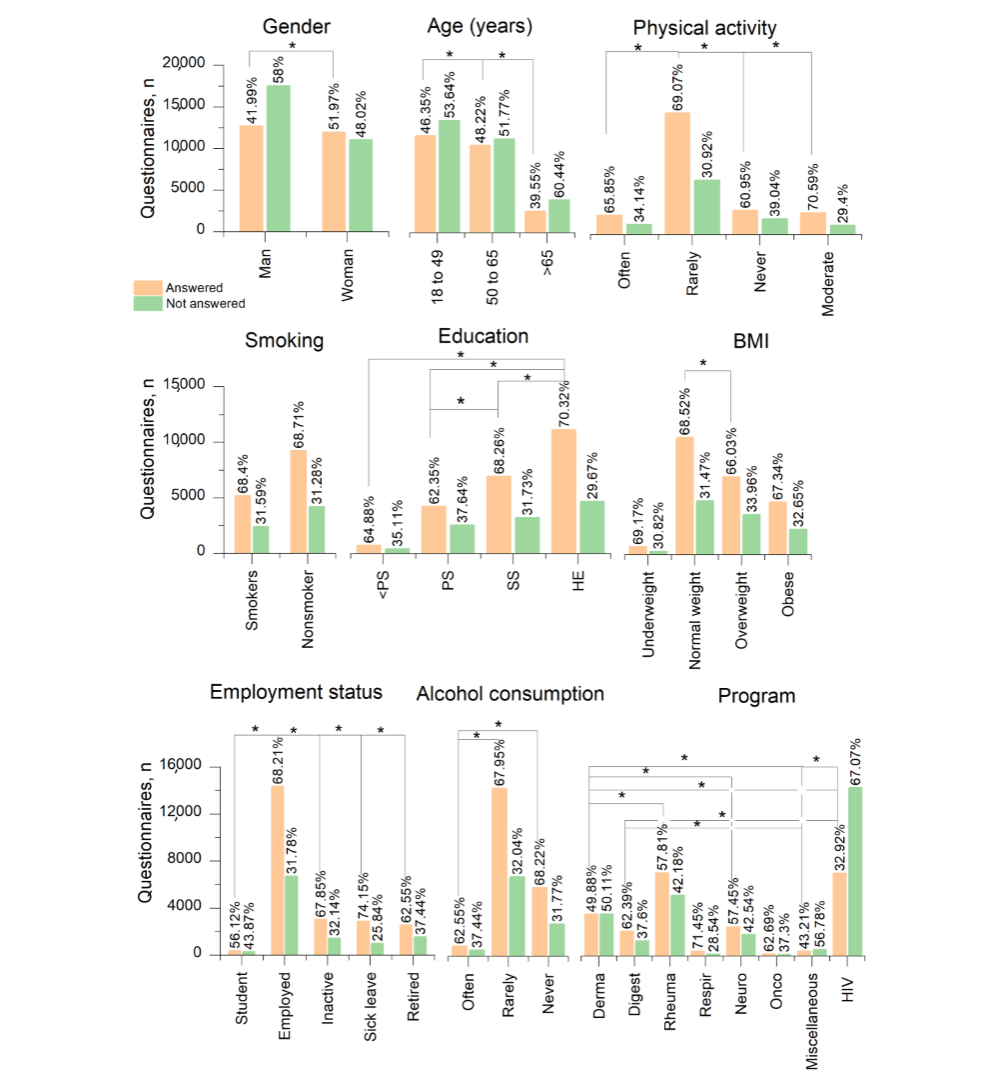
Frequencies of answered and unanswered questionnaires compared across different sociodemographic factors. *At post hoc level, the difference in the means is significant, *P*<.05. HE: higher education; PS: primary school; SS: secondary school.

Significant differences were found for gender, age, education level, employment status, alcohol consumption, and physical activity. Women were more likely to complete questionnaires than men (*P*<.001), and patients aged >65 years had a lower response rate than younger patients (*P*<.001). Higher education correlated with a higher frequency of completed questionnaires compared to the categories of preschool education (*P*<.001), primary school education (*P*<.001), and secondary school education (*P*=.002). Regarding employment status, patients on sick leave had the highest response rate (*P*<.001), and students had the lowest response rate (*P*<.001) compared to the other categories. Finally, when comparing the categories of alcohol consumption and physical activity, a lower frequency of completed questionnaires was found for frequent alcohol consumption (*P*<.001) and no physical activity (*P*<.001; Figure 9).

For disease type, significant differences among the categories were observed (Figure 9; Table S4 in Multimedia Appendix 1; refer to Table S5 in Multimedia Appendix 1 for frequencies of answered and unanswered questionnaires by disease program). The highest response rates were found for Naveta Respir, Naveta Digest, Naveta Rheuma, and Naveta Onco; and the lowest response rates were associated with the patients who had tested positive for HIV infection and those included in Naveta Miscellaneous.

In terms of satisfaction with the telemonitoring program via Naveta, 87.7% (381/434) of the patients rated their experience with scores between 7 and 10 (n=197, 45.4% provided a rating of 9 or 10; and n=184, 42.3% provided a rating of 7 or 8). The analysis in the different clinical sets did not show significant differences among them in the level of satisfaction (*P*=.63). A third of the most satisfied patients (61/197, 30.9%; rating of 9 or 10) belonged to the group categorized as Naveta Rheuma.

## Discussion

### Principal Findings

The Naveta initiative aims to promote the principles of VBHC and improve the quality of care for people with chronic diseases through the systematic integration of ePROMs and ePREMs into clinical practice settings and a continuous learning process based on result analysis. The routine collection of PROMs and PREMs enables health care providers to assess treatment effectiveness, identify unrecognized issues, and positively impact patient management and satisfaction [41-43]. High response rates to PROMs have been associated with better health outcomes and are important to ensure that the information collected is representative [21,44]. The overall response rate to ePROMs and ePREMs during the first 2.5 years of Naveta was 46.12% (24,704/53,364), with a baseline rate of 53.33% (7198/13,496). In countries with a long tradition of systematic PROM collection, such as the United Kingdom, Denmark, and the United States, response rates might range from 50% to 80% or higher [45-49]. However, comparing response rates among studies is challenging due to the lack of a standardized calculation formula [44,48]. Understanding the variables that influence these rates is fundamental to developing effective strategies to improve patient engagement. We consider that with the publication of similar studies and with the implementation of these systems in more regions, comparable data will be available, and cutoff points could be established. The association of response rate with common clinical surrogate markers would also be necessary.

These data could also be useful in future studies to assess the adequacy of PROMs and PREMs for different profiles of patients and their usefulness in clinical practice and future integration in health services databases.

Our study identified several sociodemographic factors that seemed to negatively impact response rates, including male gender, older age, lower education level, frequent alcohol consumption, being a student, and no physical activity. Women in our cohort exhibited higher response rates than men, a pattern already observed in other studies, although not consistently [44,50]. Further investigation is warranted to confirm this observation and to determine why men may be less motivated to answer health questionnaires. Older age was also significantly associated with lower response rates compared to younger age, and this is a common pattern in previous studies [44,50,51]. Possible reasons for this association include lower levels of digital confidence and skills as well as language and literacy skills, visual impairment, and health problems [50,51]. The higher response rates observed among individuals with higher levels of education in our study align with those in other studies examining barriers to completing ePROMs [52,53].

Lower education levels are often associated with lower health literacy and digital skills [52]. To the best of our knowledge, limited research has been conducted on the impact of employment status, physical activity, and alcohol consumption on PREM and PROM response rates [54,55].

The potential for bias in response rates associated with certain sociodemographic characteristics should be given due consideration and thoroughly examined to implement appropriate compensatory measures. To make the use of PROMs more accessible to a wider range of people, particularly to older and less educated patients, it could be necessary to offer some assistance (from a nurse, data manager, family members, or caregiver). This assistance could help, for example, to complete electronic questionnaires and promote a better understanding of the associated benefits [19,53].

It should be noted that, according to the study by Eriksen et al [21], PROMS equip patients with an improved understanding of their conditions, treatment, and health and increase their awareness of psychosocial issues and symptoms by encouraging disease-related reflections. However, in some cases, particularly patients with some complex conditions, the anxiety stemming from reporting symptoms or being reminded of their deteriorating health status can be a reason for not participating in such health-related survey [56].

Patients’ increased access to, and accumulation of, knowledge through digital platforms allow mobilizing and empowering them and might affect their expectations and their political engagement related to the quality of health care [57].

Naveta’s scientific committee has so far selected 20 standard sets for different chronic diseases, comprising 70 different PROMs and PREMs. Significant variations in response rates have been observed among different types of chronic conditions, with the highest rates being for respiratory, digestive, rheumatic, and oncologic diseases and the lowest rates being for HIV infection. Studies comparing response rates to ePROMs across different chronic diseases, especially when using condition-specific standard sets of questionnaires, are scarce. In a study describing the implementation of the WestChronic PRO system in Denmark, which included 22 PRO projects for 18 chronic diseases, the highest response rates were observed in patients with epilepsy and prostate cancer, whereas the lowest rates were observed in patients with stroke [58]. A review of response rates available in clinical quality registries and databases that collect PROMs for all types of health conditions found that patients with chronic conditions and cancer have the highest response rates at baseline [48]. The differences observed in response rates in our study may be related to the nature of the specific conditions. However, it would be necessary to determine the role of disease-specific PROMs and other factors such as the severity of symptoms and the presence of comorbidities [59,60]. Further research is necessary to fully understand these differences and to implement strategies to improve response rates across pathologies.

During the first 6 months of follow-up, response rates declined across all disease types, except in the case of the group of patients with oncologic disease, where a notable increase was observed. Subsequently, the overall response rate increased and approached baseline levels, and the response rate pattern in the group of patients with oncologic disease became comparable to the response rate patterns in the other groups. It should be noted that the number of questionnaires that patients were requested to complete varied over time in each group. This initial increase in response rates in patients with oncologic disease has not been described in other studies and should be investigated in a larger cohort to elucidate potential underlying causes [49,61,62]. One possible explanation is the support provided by the cancer functional units, which include multidisciplinary professionals such as oncologists, surgeons, pharmacists, nurses, psychologists, nutritionists, and physiotherapists; nurses, in particular, play an important role in motivating patients to respond to PROMs. However, as side effects become more pronounced or accumulate over time, patient motivation may diminish.

The overall decline in response rates at follow-up is a common tendency in numerous studies, with only a few exceptions [44,48,49,58,60,63,64]. This decline has been attributed to survey fatigue, which is associated with factors such as response burden, questionnaire length, perceived item irrelevance, older age, disease severity, and the presence of comorbidities [59,64,65]. When selecting standard sets and the frequency of questionnaire administration for each pathology, it is crucial to balance the need to obtain comprehensive information with the potential burden on patients if they have to complete numerous questionnaires. To address survey fatigue and enhance response rates, Naveta’s scientific committee is currently exploring new strategies inspired by the PROMIS Computer Adaptive Tests and the studies that have investigated patient burden [59,65-67]. These strategies include reducing the number of PROMs in standard sets; labeling some of them as optional; using shorter questionnaires; and reducing, delaying, or spreading over time the administration of questionnaires.

Unlike the traditional use of PROMs in clinical practice as a supplement to the patient’s follow-up, PROM-based follow-up with AmbuFlex and Naveta platforms represents a new model of service delivery where PROMs are used as the basis for outpatient follow-up [68].

Implementing the routine collection of PROMs and PREMs in clinical settings involves integrating technological systems for electronic questionnaire administration [69]. The advantages of ePROMs and ePREMs over paper questionnaires include reduced data entry errors, shorter completion times, real-time results, improved symptom management, the prioritization of clinical visits, and cost reduction [19,70]. In addition, automated alerts enable health care professionals to promptly address serious complications, reducing symptom burden and the risk of hospital readmissions [19]. Challenges associated with electronic questionnaires include privacy concerns, a higher initial investment in infrastructure, and potential bias due to patients’ lack of digital literacy [19].

Positive feedback from patients regarding PROM monitoring via the Naveta system was demonstrated by the results of the satisfaction questionnaire. Aligned to our commitment to improve the health and well-being of patients with chronic diseases and guided by the principles of VBHC and right care [8,9], our initiative aims to ensure that digitalization goes hand in hand with humanization. Examples of this approach include health care professionals personally contacting patients in response to flagged alerts identified through questionnaire results or establishing communication channels for consultations. Recently, to help bridge the digital gap, data managers have been introduced to contact patients who do not complete questionnaires. Their role involves understanding the reasons for noncompletion, clarifying doubts, emphasizing the benefits of completing PROMs, and offering assistance to respond if desired. These actions reinforce our commitment to a patient-centered model that embraces technology while reaffirming the human connection.

Moreover, we believe that it is important to involve patients in the design of health technology. Effective patient engagement can profoundly change how patient-centered research is conceptualized and conducted, resulting in better patient-centered care, management, and measurement [71]. In our case, the feedback obtained in the satisfaction surveys is taken into consideration for making improvements to the Naveta platform. While, currently, the perspective of patients is indirectly obtained through contacts with patients’ associations, we plan in the future to include patient representatives in the meetings of the scientific committee.

### Strengths and Limitations

This study provides empirical evidence on the implementation of a telemonitoring system for patients who are chronically ill through electronic questionnaires at different hospitals and in different regions in Spain. Our comprehensive examination of multiple sociodemographic factors that influence the response rate to ePROMs and ePREMs provides valuables insights into existing literature. This study is one of the few that has compared response rates to ePROMs across different types of chronic diseases when specific standard sets of questionnaires were administered for each disease. Our findings contribute to the current understanding of the strategies needed to implement the systematic collection of ePROMs and ePREMs for patients with chronic conditions in clinical practice according to the principles of value-based care. We present a feasible model of telemedicine, which allows the remote clinical follow-up of a high number of patients with chronic conditions, helped by real-time patient-reported measures, that could substantially change the basis of current clinical management and facilitate shared decision-making, with substantial improvements in comparison with traditional on-site care.

However, 2 limitations should be noted. First, the sample size in certain disease categories (eg, oncologic diseases) was relatively small due to the recent introduction and limited adoption of the Naveta initiative in certain settings. The low number of patients in certain disease groups and the high number of missing values in certain sociodemographic categories reduced the statistical power of the study and did not allow us to perform more specific statistical analysis. Second, the retrospective design of the observational study meant that certain variables, such as the total number of questionnaires and variations in the frequency of questionnaire administration, were not systematically controlled in the analysis of the effect of different diseases on response rates and their evolution.

### Conclusions

In conclusion, the overall response rate to the questionnaires during the first 2.5 years of the Naveta initiative was 46.12% (24,704/53,364), with several sociodemographic factors associated with lower response rates. There was a general trend of decreasing rates in the first 6 months, possibly indicating survey fatigue. Recognizing the impact of sociodemographic and clinical factors on response rates may help identify barriers for certain patient groups to participate in telemonitoring programs, suggesting the need to tailor telemonitoring approaches according to patient populations.

## Appendix

Multimedia Appendix 1 Patients’ clinical characteristics and response pattern.

## Abbreviations

ePREM: electronic patient-reported experience measure
ePROM: electronic patient-reported outcome measure
FARUPEIB: Association of Pharmacists of Outpatient Departments of the Balearic Islands
ICHOM: International Consortium for Health Outcomes Measurement
P3CEQ: Person-Centered Coordinated Care Experience Questionnaire
PREM: patient-reported experience measure
PRO: patient-reported outcome
PROM: patient-reported outcome measure
PROMIS: Patient-Reported Outcomes Measurement Information System
PROTEUS: Patient-Reported Outcomes Tools: Engaging Users and Stakeholders
SIAQ: Self-Injection Assessment Questionnaire
VBHC: value-based health care
