# Supplementary material for: Implementing Systematic Patient-Reported Measures for Chronic Conditions Through the Naveta Value-Based Telemedicine Initiative: Observational Retrospective Multicenter Study
Source: JMIR Mhealth Uhealth. 2024 Jun 28;12:e56196. doi: 10.2196/56196 (PMC11245666; doi:10.2196/56196)
Supplement: Multimedia Appendix 1 [file mhealth_v12i1e56196_app1.docx]

**Supplemental material:**

**Table S1.** Number of patients in each disease program and disease type category.

| **Disease type category** | **N** | **Disease program** | **n (%)** |
| --- | --- | --- | --- |
| **Skin diseases (Naveta Derma)** | 414 |  |  |
|  |  | Atopic dermatitis | 52 (12.56) |
|  |  | Psoriasis | 312 (75.36) |
|  |  | Urticaria | 24 (5.80) |
|  |  | Hidradenitis suppurativa | 26 (62.80) |
| **Gastrointestinal diseases (Naveta Digest)** | 145 |  |  |
|  |  | Ulcerative Colitis | 30 (20.69) |
|  |  | Crohn's | 115 (79.31) |
| **Respiratory diseases (Naveta Respir)** | 22 |  |  |
|  |  | Asthma | 22 (100) |
| **Neurological diseases (Naveta Neuro)** | 209 |  |  |
|  |  | Multiple Sclerosis | 96 (45.93) |
|  |  | Migraine | 113 (54.07) |
| **Oncologic diseases (Naveta Onco)** | 28 |  |  |
|  |  | Breast Cancer | 17 (60.71) |
|  |  | Prostate Cancer | 9 (32.14) |
|  |  | Lung Cancer | 2 (7.14) |
| **Other diseases (Naveta Miscellaneous)** | 372 |  |  |
|  |  | Anemia | 79 (58.96) |
|  |  | Ophthalmological diseases | 55 (41.04) |
| **Rheumatic diseases (Naveta Rheuma)** | 372 |  |  |
|  |  | Psoriatic Arthritis | 120 (32.26) |
|  |  | Rheumatoid Arthritis | 123 (33.06) |
|  |  | Ankylosing Spondylitis | 129 (34.68) |
| **HIV positive (Naveta HIV)** | 2083 |  |  |
|  |  | HIV-positive | 2083 (100) |

**Table S2**. Sociodemographic characteristics of study participants and their distribution by type of disease^a^.

|  | **Total^b^** | **Naveta**  **Derma** | **Naveta**  **Digest** | **Naveta**  **Rheuma** | **Naveta**  **Respir** | **Naveta**  **Neuro** | **Naveta**  **Onco** | **Naveta Miscellaneous** | **Naveta**  **HIV** |
| --- | --- | --- | --- | --- | --- | --- | --- | --- | --- |
| **Age** |  |  |  |  |  |  |  |  |  |
| N | 3372 | 414 | 145 | 372 | 22 | 209 | 28 | 134 | 2083 |
| Years, mean (SD) | 50,36 (12,91) | 47,01 (14,17) | 44,36 (13,37) | 53,30 (13,31) | 52,59 (11,58) | 46,15 (10,53) | 57,57 (14,03) | 65,92 (15,12) | 50,22 (11,59) |
| **Gender** |  |  |  |  |  |  |  |  |  |
| N | 3372 | 414 | 145 | 372 | 22 | 209 | 28 | 134 | 2083 |
| Women, n (%) | 1234 (36,6) | 202 (48,79) | 63 (43,45) | 228 (61,29) | 19 (86,36) | 158 (75,6) | 17 (60,71) | 73 (54,48) | 487 (23,38) |
| Men, n (%) | 2128 (63,11) | 211 (50,97) | 82 (56,55) | 144 (38,71) | 3 (13,64) | 50 (23,92) | 11 (39,29) | 61 (45,52) | 1588 (76,24) |
| Others, n (%) | 10 (0,3) | 1 (0,24) | 0 (0) | 0 (0) | 0 (0) | 1 (0,48) | 0 (0) | 0 (0) | 8 (0,38) |
| **BMI** |  |  |  |  |  |  |  |  |  |
| N | 1888 | 295 | 112 | 315 | 20 | 159 | 20 | 84 | 908 |
| kg/m^2^, mean (SD) | 25,97 (4,95) | 27,74 (5,66) | 24,74 (5,10) | 27,14 (5,70) | 26,57 (5,62) | 24,77 (5,40) | 25,71 (4,12) | 27,72 (4,73) | 25,17 (3,96) |
| **Educational level** |  |  |  |  |  |  |  |  |  |
| N | 1889 | 293 | 114 | 312 | 20 | 159 | 20 | 84 | 912 |
| Preschool education, n (%) | 66 (3,49) | 6 (2,05) | 6 (5,26) | 13 (4,17) | 2 (10) | 4 (2,52) | 2 (10) | 15 (17,86) | 20 (2,19) |
| Primary school, n(%) | 381 (20,17) | 46 (15,7) | 22 (19,3) | 87 (27,88) | 4 (20) | 29 (18,24) | 1 (5) | 29 (34,52) | 169 (18,53) |
| Secondary school, n (%) | 545 (28,85) | 94 (32,08) | 29 (25,44) | 88 (28,21) | 4 (20) | 40 (25,16) | 7 (35) | 17 (20,24) | 273 (29,93) |
| Higher education, n (%) | 897 (47,49) | 147 (50,17) | 57 (50) | 124 (39,74) | 10 (50) | 86 (54,09) | 10 (50) | 23 (27,38) | 450 (49,34) |
| **Employment status** |  |  |  |  |  |  |  |  |  |
| N | 1889 | 293 | 114 | 312 | 20 | 159 | 20 | 84 | 912 |
| Student, n (%) | 32 (1,69) | 9 (3,07) | 7 (6,14) | 2 (0,64) | 0 (0) | 4 (2,52) | 0 (0) | 0 (0) | 10 (1,1) |
| Employed, n (%) | 1165 (61,67) | 196 (66,89) | 84 (73,68) | 180 (57,69) | 10 (50) | 102 (64,15) | 4 (20) | 19 (22,62) | 585 (64,14) |
| Unemployed, n (%) | 262 (13,87) | 43 (14,68) | 10 (8,77) | 32 (10,26) | 3 (15) | 17 (10,69) | 0 (0) | 4 (4,76) | 154 (16,89) |
| On sick leave, n (%) | 192 (10,16) | 18 (6,14) | 7 (6,14) | 44 (14,1) | 2 (10) | 28 (17,61) | 8 (40) | 9 (10,71) | 82 (8,99) |
| Retired, n (%) | 238 (12,6) | 27 (9,22) | 6 (5,26) | 54 (17,31) | 5 (25) | 8 (5,03) | 8 (40) | 52 (61,9) | 81 (8,88) |
| **Smoking habit** |  |  |  |  |  |  |  |  |  |
| N | 1112 | 494 | 167 | 438 | 29 | 269 | 27 | 8 | 1500 |
| Smokers, n (%) | 403 (36,8) | 68 (13,77) | 25 (14,97) | 34 (7,76) | 0 (0) | 33 (12,27) | 5 (18,52) | 0 (0) | 243 (16,2) |
| Non-smokers, n (%) | 692 (63,2) | 133 (26,92) | 32 (19,16) | 90 (20,55) | 9 (31,03) | 79 (29,37) | 4 (14,81) | 8 (100) | 350 (23,33) |
| **Alcohol consumption** |  |  |  |  |  |  |  |  |  |
| N | 1906 | 293 | 110 | 314 | 20 | 157 | 18 | 84 | 907 |
| Frequent, n (%) | 88 (4,69) | 10 (3,41) | 6 (5,45) | 17 (5,41) | 0 (0) | 5 (3,18) | 2 (11,11) | 3 (3,57) | 46 (5,07) |
| Rare, n (%) | 1277 (68) | 214 (73,04) | 72 (65,45) | 203 (64,65) | 12 (60) | 103 (65,61) | 14 (77,78) | 39 (46,43) | 637 (70,23) |
| Never, n (%) | 513 (27,32) | 69 (23,55) | 32 (29,09) | 94 (29,94) | 8 (40) | 49 (31,21) | 2 (11,11) | 42 (50) | 224 (24,7) |
| **Physical activity** |  |  |  |  |  |  |  |  |  |
| N | 1882 | 291 | 109 | 308 | 20 | 157 | 19 | 84 | 893 |
| Frequent (%) | 215 (11,58) | 28 (9,62) | 9 (8,26) | 28 (9,09) | 1 (5) | 10 (6,37) | 1 (5,26) | 8 (9,52) | 136 (15,23) |
| Moderate, n (%) | 209 (11,26) | 34 (11,68) | 13 (11,93) | 31 (10,06) | 0 (0) | 11 (7,01) | 1 (5,26) | 4 (4,76) | 117 (13,1) |
| Rare, n (%) | 1188 (64,01) | 188 (64,6) | 78 (71,56) | 190 (61,69) | 14 (70) | 111 (70,7) | 16 (84,21) | 58 (69,05) | 544 (60,92) |
| No activity, n (%) | 244 (13,15) | 41 (14,09) | 9 (8,26) | 59 (19,16) | 5 (25) | 25 (15,92) | 1 (5,26) | 14 (16,67) | 96 (10,75) |

^a^ Demographic characteristics were reported as mean ± standard deviation (SD) for quantitative traits and counts (%) for qualitative variables.

^b^ 84 participants were registered for more than one chronic condition, so the categories are not mutually exclusive.

BMI: body mass index; n= number of available data for each variable; SD: standard deviation.

**Table S3**. Response rates by disease type at different time intervals.

|  | **Total** | **Naveta Derma** | **Naveta Digest** | **Naveta Rheuma** | **Naveta Respir** | **Naveta Neuro** | **Naveta Onco** | **Naveta Miscellaneous** | **Naveta**  **HIV** |
| --- | --- | --- | --- | --- | --- | --- | --- | --- | --- |
| **All intervals, CQ/SQ (%)** | 24704/53364 (46,29) | 3778/7573 (49,89) | 2247/3601 (62,4) | 7506/12982 (57,82) | 433/606 (71,45) | 2631/4579 (57,46) | 200/319 (62,7) | 436/1009 (43,21) | 7473/22695 (32,93) |
| **Baseline, CQ/SQ (%)** | 7198/13496 (53,33) | 1114/1807 (61,65) | 390/546 (71,43) | 1123/1611 (69,71) | 90/115 (78,26) | 845/1224 (69,04) | 84/129 (65,12) | 300/565 (53,1) | 3252/7499 (43,37) |
| **Until 6 months, CQ/SQ (%)** | 9131/22000 (41,5) | 1411/2892 (48,79) | 753/1199 (62,8) | 2655/4473 (59,36) | 151/216 (69,91) | 990/1852 (53,46) | 6/6  (100) | 108/401 (26,93) | 2978/10825 (27,51) |
| **Between 6 months and 1 year, CQ/SQ (%)** | 4474/10239 (43,7) | 717/1445 (49,62) | 532/839 (63,41) | 1812/3311 (54,73) | 108/179 (60,34) | 561/1165 (48,15) | 25/42 (59,52) | 9/16  (56,25) | 891/3258 (27,35) |
| **Between 1 and 2.5 years, CQ/SQ (%)** | 3901/7629 (51,13) | 536/1429 (37,51) | 572/1017 (56,24) | 1916/3587 (53,42) | 84/96 (87,5) | 235/338 (69,53) | 85/142 (59,86) | 19/27  (70,37) | 352/1113 (31,63) |

CQ: number of completed questionnaires; SQ: number of sent questionnaires.

**Table S4**. Frequencies of answered and unanswered questionnaires by disease program.

| **Disease Program** | **Number of answered** **questionnaires**  (%) | **Number of unanswered questionnaires**  (%) |
| --- | --- | --- |
|  | 17,916 | 15,574 |
| Psoriatic arthritis | 209 (46.04) | 245 (53.96) |
| Rheumatoid arthritis | 2,885 (57.24) | 2,155 (42.76) |
| Asthma | 433 (71.45) | 173 (28.55) |
| Breast cancer | 97 (55.11) | 79 (44.89) |
| Prostate cancer | 90 (69.77) | 39 (30.23) |
| Lung cancer | 13 (92.86) | 1 (7.14) |
| Ulcerative colitis | 490 (66.85) | 243 (33.15) |
| Crohn's disease | 687 (46.08) | 804 (53.92) |
| Atopic dermatitis | 1,757 (61.26) | 1,111 (38.74) |
| Multiple sclerosis | 1,098 (56.37) | 850 (43.63) |
| Spondylitis | 2,399 (63.1) | 1403 (36.9) |
| Hidradenitis suppurativa | 305 (46.28) | 354 (53.72) |
| Migraine | 1,533 (58.27) | 1,098 (41.73) |
| Ophthalmological diseases | 227 (40.9) | 328 (59.1) |
| Psoriasis | 2,591 (52.74) | 2,417 (48.26) |
| Urticaria | 195 (46.99) | 220 (53.01) |
| HIV | 7,473 (32.93) | 15,222 (67.07) |

**Table S5**. Comparison of the frequencies of answered and unanswered questionnaires by sociodemographic and clinical characteristics.

| **Socio-demographic and clinical characteristics** | | **Number of answered** **questionnaires**  **(%)** | **Number of unanswered questionnaires**  **(%)** | ***P* value** |
| --- | --- | --- | --- | --- |
| **Gender** | | 24,704 | 28,660 | **<.001** |
|  | Males | 12,696 (42) | 17,536 (58) |  |
|  | Females | 11,957 (51.97) | 11,050 (48.03) |  |
|  | Others^a^ | 51 (40.80) | 74 (59.20) |  |
| **Age**^b^ | | 24,704 | 28,660 | **<.001** |
|  | 18-49 years | 11,634 (46.35) | 13,464 (53.65) |  |
|  | 50-65 years | 10,503 (48.23) | 11,274 (51.77) |  |
|  | >65 years | 2,567 (39.56) | 3,922 (60.44) |  |
| **BMI**^c^ | | 23,106 | 11,113 | **<.001** |
|  | Underweight | 716 (69.18) | 319 (30.82) |  |
|  | Healthy weight | 10,606 (68.52) | 4,872 (31.48) |  |
|  | Overweight | 7,033 (66.03) | 3,618 (33.97) |  |
|  | Obesity | 4,751 (67.34) | 2,304 (32.66) |  |
| **Educational level**^d^ | | 23,168 | 10,941 | **<.001** |
|  | Preschool education | 754 (64.89) | 408 (35.11) |  |
|  | Primary school | 4,270 (62.35) | 2,578 (37.65) |  |
|  | Secondary school | 6,962 (68.27) | 3,236 (31.73) |  |
|  | Higher education | 11,182 (70.32) | 4,719 (29.68) |  |
| **Employment status**^e^ | | 14,728 | 6,981 | **<.001** |
|  | Student | 371 (56.13) | 290 (43.87) |  |
|  | Employed | 14,357 (68.21) | 6,691 (31.79) |  |
|  | Unemployed | 3,021 (67.86) | 1,431 (32.14) |  |
|  | On sick leave | 2,858 (74.16) | 996 (24.89) |  |
|  | Retired | 2,561 (62.55) | 1,533 (37.45) |  |
| **Smoking status** | | 14,469 | 6622 | .65 |
|  | Smokers | 5,204 (68.40) | 2,404 (31.60) |  |
|  | Non-smokers | 9,265 (68.72) | 4,218 (31.28) |  |
| **Alcohol consumption**^d^ | | 22,951 | 10,903 | **<.001** |
|  | Often | 922 (62.55) | 552 (37.45) |  |
|  | Rarely | 15,636 (67.95) | 7,374 (32.05) |  |
|  | Never | 6,393 (68.23) | 2,977 (31.77) |  |
| **Physical activity**^f^ | | 22,544 | 10,742 | **<.001** |
|  | Frequent | 2,349 (65,85) | 1,218 (34.15) |  |
|  | Moderate | 2,639 (70.6) | 1,099 (29.4) |  |
|  | Rare | 14,621 (69.08) | 6,545 (30.92) |  |
|  | No activity | 2,935 (60.96) | 1,880 (39.04) |  |
| **Disease type**^g^ | | 24,704 | 28,660 | **<.001** |
|  | Naveta Derma | 3,778 (49.89) | 3,795 (50.11) |  |
|  | Naveta Digest | 2,247 (62.40) | 1,354 (37.60) |  |
|  | Naveta Rheuma | 7,506 (57.82) | 5,476 (42.18) |  |
|  | Naveta Respir | 433 (71.45) | 173 (28.55) |  |
|  | Naveta Neuro | 2,631 (57.46) | 1,948 (42.54) |  |
|  | Naveta Onco | 200 (62.70,) | 119 (37.30) |  |
|  | Naveta HIV | 7473 (32.93) | 15,222 (67.07) |  |
|  | Naveta Miscellaneous | 436 (43.21) | 573 (56.79) |  |

^a^ This category was not included in the analysis because of its low frequency.

^b^ All pairwise comparisons across age categories were statistically significant (**adjusted *P*<.001**.)

^c^ Pairwise comparisons across BMI categories: between underweight and healthy weight (adjusted *P*=.97); underweight and overweight (adjusted *P* =.16); underweight and obesity (adjusted *P*=.62); **healthy weight and overweight** (**adjusted  *P* <.001**); healthy weight and obesity (adjusted *P*=.27); and overweight and obesity (adjusted *P*=.25).

^d^ Pairwise comparisons across education categories: between preschool education and primary school (adjusted *P*=.33); preschool education and secondary school (adjusted *P*=.08); **preschool education and higher education** (**adjusted *P*<.001**); **primary school and secondary school** (**adjusted *P*<.001**); **primary school and higher education** (adjusted *P*<.001); and **secondary and higher education** (**adjusted *P*=.002**).

^e^ Pairwise comparisons across employment status categories were statistically significant (**adjusted *P*<.001**), except between employed and unemployed (adjusted *P*=.99).

^d^ Pairwise comparisons across alcohol consumption categories: between **often and rarely** (**adjusted *P*<.001**); **often and never** (**adjusted *P*<.001**); and rarely and never (adjusted *P=*.87).

^f^ Pairwise comparisons across all physical activity categories were statistically significant (**adjusted *P*<.001**), except between moderate and rarely (adjusted *P*=.24).

^g^ Pairwise comparisons across disease type categories: between **Derma and Digest** (**adjusted *P*<.001**); **Derma and HIV** (**adjusted *P*<.001**); **Digest and HIV** (**adjusted *P*<.001**); **Derma and Miscellaneous** (**adjusted *P*=.001**); **Digest and Miscellaneous** (**adjusted *P*<.001**); **HIV and Miscellaneous** (**adjusted *P*=.001**); **Derma and Neuro** (**adjusted *P*<.001**); **Digest and Neuro** (**adjusted *P*<.001**); **HIV and Neuro** (**adjusted *P*<.001**); **Miscellaneous and Neuro** (**adjusted *P*<.001**); **Derma and Onco** (**adjusted *P*<.001**); Digest and Onco (adjusted *P>.99*); **HIV and Onco** (**adjusted *P*< .001**); **Miscellaneous and Onco** (**adjusted *P*<.001**); Neuro and Onco (adjusted *P*>.99); **Derma and Respir** (**adjusted *P*<.001**); **Digest and Respir** (**adjusted *P*<.001**); **HIV and Respir** (**adjusted *P*<.001**); **Miscellaneous and Respir** (**adjusted *P*<.001**); **Neuro and Respir** (**adjusted *P* <.001**); Onco and Respir (adjusted *P*=.093); **Derma and Rheuma** (**adjusted *P*<.001**); **Digest and Rheuma** (**adjusted *P*<.001**); **HIV and Rheuma** (**adjusted *P*<.001**); **Miscellaneous and Rheuma** (**adjusted *P*<.001**); Neuro and Rheuma (adjusted *P*=0.99); Onco and Rheuma (adjusted *P*=0.61); and Respir and Rheuma (adjusted *P*<.001).

BMI: body mass index; N: number of available data for each variable.
